# Reversible Hyperpolarization of Ketoisocaproate Using Sulfoxide‐containing Polarization Transfer Catalysts

**DOI:** 10.1002/cphc.202000825

**Published:** 2020-11-26

**Authors:** Ben. J. Tickner, Fadi Ahwal, Adrian C. Whitwood, Simon B. Duckett

**Affiliations:** ^1^ Centre for Hyperpolarisation in Magnetic Resonance University of York, Heslington York U.K. YO10 5NY; ^2^ Department of Chemistry University of York, Heslington York U.K. YO10 5DD; ^3^ NMR Research Unit, Faculty of Science University of Oulu P.O. Box 3000 90014 Oulu Finland

**Keywords:** iridium dimers, ketoisocaproate, *para*hydrogen, SABRE hyperpolarization, sulfoxides

## Abstract

The substrate scope of sulfoxide‐containing magnetisation transfer catalysts is extended to hyperpolarize *α*‐ketoisocaproate and *α*‐ketoisocaproate‐1‐[^13^C]. This is achieved by forming [Ir(H)_2_(*κ* ^2^‐ketoisocaproate)(*N*‐heterocyclic carbene)(sulfoxide)] which transfers latent magnetism from *p*‐H_2_ *via* the signal amplification by reversible exchange (SABRE) process. The effect of polarization transfer field on the formation of enhanced ^13^C magnetization is evaluated. Consequently, performing SABRE in a 0.5 μT field enabled most efficient magnetisation transfer. ^13^C NMR signals for *α*‐ketoisocaproate‐1‐[^13^C] in methanol‐*d*_4_ are up to 985‐fold more intense than their traditional Boltzmann derived signal intensity (0.8 % ^13^C polarisation). Single crystal X‐ray diffraction reveals the formation of the novel catalyst decomposition products [Ir(*μ*‐H)(H)_2_(IMes)(SO(Ph)(Me)_2_)]_2_ and [(Ir(H)_2_(IMes)(SO(Me)_2_))_2_(*μ*‐S)] when the sulfoxides methylphenylsulfoxide and dimethylsulfoxide are used respectively.

## Graphical abstract

**Extending substrate scope**: It is demonstrated that the scope of sulfoxide‐containing SABRE polarization transfer catalysts can be extended to hyperpolarize molecules with similar α‐keto acid motifs to pyruvate. This could lead to a larger range of potential applications of SABRE in monitoring chemical transformations.

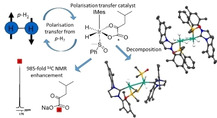

The detection of molecular species with a low concentration (<1 mM), or a transient nature (lifetime<1 s), using Magnetic Resonance (MR) can be extremely challenging. This limitation stems from low MR sensitivity, which is related to low Boltzmann population differences between the nuclear spin energy levels interrogated by MR radio frequency excitation. Hyperpolarization can be used to improve sensitivity by generating non‐Boltzmann population distributions within nuclear spin states and consequently yields MR signals that can be several orders of magnitude larger than those usually achieved.^[1]^

Of the many hyperpolarization methods available, those based on *para*‐hydrogen (*p*‐H_2_) are straightforward to utilize.[^1^, ^2^] This is due to simple isolation of hydrogen gas enriched in its *para* form (up to 99 %) by cooling to low temperatures (28 K) in the presence of a spin exchange catalyst.^[3]^ The *para‐*isomer enrichment survives subsequent warming once it is no longer in the presence of the catalyst. It has been known since the 1980s that the spin order associated with *p*‐H_2_ can be transferred into other molecules after involvement in a symmetry breaking reaction.[^4^, ^5^, ^6^] Initially, this was achieved *via* the hydrogenation of a target organic or metal complex and the MR signals of those product protons previously located in *p*‐H_2_ were significantly hyperpolarized as a consequence.[^4^, ^5^, ^6^]

More recently, a non‐hydrogenative approach termed signal amplification by reversible exchange (SABRE) has been described which transfers *p*‐H_2_ spin order to molecules in a process that does not involve chemically changing the target molecule.^[7]^ In SABRE, the symmetry of *p*‐H_2_ is first broken through a reversible oxidative addition reaction to a metal centre. Subsequent transfer of polarization from *p*‐H_2_ derived hydride ligands to other molecules ligated to the metal centre then proceeds through the resulting *J*‐coupled network of the metal catalyst.^[8]^ This transfer can occur spontaneously at low magnetic fields (mT^[9]^ or μT^[10]^ for transfer to ^1^H or heteronuclear sites respectively). Transfer can also be driven by radiofrequency excitation at high magnetic field (T).^[11]^ Dissociation of the target molecule releases hyperpolarized ligands into free solution. The proportion of ligand that exists in this non‐equilibrium spin state increases with *p*‐H_2_ shaking time in accordance with the catalytic nature of this process.^[7]^ However, the enhanced MR signals for these systems will eventually decay due to relaxation effects and consumption of the *p*‐H_2_ feedstock.

Many researchers have optimized the polarization transfer catalysts that are essential for SABRE hyperpolarization.[^9^, ^12^, ^13^, ^14^, ^15^, ^16^, ^17^, ^18^, ^19^, ^20^, ^21^, ^22^] Nowadays, iridium catalysts based on N‐heterocyclic carbenes (NHCs) are typically used in preference to the phosphine containing catalysts used in early developments.[^23^, ^24^] Since then, the performance of NHC‐based catalysts has been optimized by using the steric and electronic properties of the NHC to tune substrate exchange kinetics[^12^, ^22^] and relaxation effects.^[21]^ Structural modifications of the SABRE catalyst have been used as a route to facilitate ligation, exchange, and hyperpolarization of sterically bulky substrates[^12^, ^19^] or even to facilitate hyperpolarization in aqueous solvents.^[25]^ Most of these Iridium‐NHC catalysts are of the form [Ir(H)_2_(NHC)(substrate)_3_]Cl and while they are capable of delivering NMR signal gains for ^1^H,[^9^, ^21^, ^22^] ^15^N,[^10^, ^17^, ^26^] ^13^C,[^27^, ^28^] ^19^F,[^29^, ^30^] ^31^P^[31]^ and other nuclear sites^[32]^ in a range of molecules including N‐heterocycles,[^10^, ^14^, ^17^, ^18^, ^19^, ^20^, ^21^, ^22^, ^23^, ^24^, ^32^] nitriles,^[33]^ amines^[34]^ and many others,[^35^, ^36^] the substrate scope of SABRE is most commonly molecules with N‐donor sites that readily ligate to iridium.

Recently, these restrictions are being lifted through the use of relayed polarization transfer (SABRE‐Relay)[^37^, ^38^, ^39^, ^40^, ^41^, ^42^] which allows polarization of molecules such as alcohols,^[38]^ sugars,^[39]^ silanols^[40]^ and others^[41]^ without their direct ligation to iridium. Similarly, the hyperpolarization of weakly donating O‐donor molecules such as pyruvate^[43]^ and acetate^[44]^ has been demonstrated. In the case of pyruvate, this proved to be possible by using sulfoxide containing iridium‐NHC catalysts of the form [Ir(H)_2_(*κ* ^2^‐pyruvate)(NHC)(sulfoxide)], as depicted in Scheme 1.[^16^, ^43^] Mechanistic studies on these systems have reported that [IrCl(H)_2_(NHC)(sulfoxide)_2_] plays a role in refreshing the *p*‐H_2_ spin order needed for SABRE catalysis.[^43^, ^45^] Optimization has achieved ^13^C NMR signal gains of up to 2135‐fold (1.7 % ^13^C polarisation) for sodium pyruvate‐1,2‐[^13^C_2_] in methanol‐*d*_4_ when the NHC is IMes (where IMes is 1,3‐bis(2,4,6‐trimethylphenyl)imidazole‐2‐ylidene) and the sulfoxide is methylphenylsulfoxide.^[16]^ These sulfoxide‐containing catalysts have been applied to the hyperpolarization of molecules such as oxalate, although in this case, the formation of oxalate bridged iridium dimers of the form [(Ir(H)_2_(NHC)(sulfoxide))_2_(*μ*,1*κ* ^2^,2*κ* ^2^‐oxalate)] was indicated which restricted the necessary ligand exchange processes required for SABRE.^[45]^

**Scheme 1:**
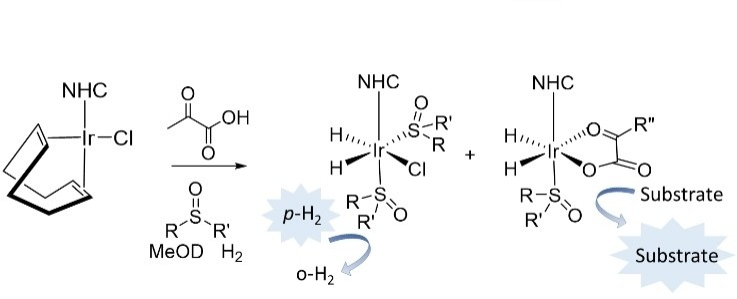
Sulfoxide‐containing polarization transfer catalysts of the form [Ir(H)_2_(*κ* ^2^‐pyruvate)(NHC)(sulfoxide)] (in the case where R” is CH_3_) are synthesized by reaction of an [IrCl(*cis*,*cis*‐1,5‐cyclooctadiene)(NHC)] precatalyst

In this work, we apply these novel sulfoxide‐containing magnetization transfer catalysts to molecules with similar *α*‐keto acid moieties to pyruvate. We demonstrate that these catalysts can deliver significant ^13^C NMR signal gains for ketoisocaproate (KIC), a common metabolic precursor for the amino acid leucine that has been used to monitor amino acid metabolism *in vivo*.[^46^, ^47^, ^48^]

Our work begins by investigating if analogous polarization transfer catalysts of the form [Ir(H)_2_(*κ* ^2^‐KIC)(NHC)(sulfoxide)] can be created. Therefore, a solution containing [IrCl(COD)(IMes)] (5 mM), ketoisocaproate (KIC) (35 mM) and dimethyl sulfoxide (22.5 mM) was activated with 3 bar H_2_ in methanol‐*d*_4_ (0.6 mL). Thermal ^1^H NMR measurements were then recorded to examine whether any hydride containing complexes were formed. A major product, with hydride ligands resonating at *δ=*−27.09 and −29.19 ppm, proved to form which is consistent with [Ir(H)_2_(*κ* ^2^‐KIC)(dimethylsulfoxide)(IMes)] in which the hydride ligands and ligated KIC occupy the same plane (Figure 1a). This solution was shaken for 10 seconds with *p*‐H_2_ (3 bar) at 6.5 mT before a single scan ^1^H NMR spectrum was recorded at 9.4 T. The integral intensities of these two hydride ligand signals were found to be 700‐times larger than their normal values (Figure 1c). Weakly hyperpolarized signals for [IrCl(H)_2_(dimethylsulfoxide)_2_(IMes)][^43^, ^45^] at *δ=*−21.68 and −15.73 ppm were also observed in addition to resonances at *δ=*−15.09 and −24.22 ppm. The latter signals are expected to correspond to the isomer of [Ir(H)_2_(*κ* ^2^‐KIC)(dimethylsulfoxide)(IMes)] in which the KIC and hydride ligands are located as detailed in Figure 1.^[43]^ Weakly hyperpolarized signals for the ^1^H resonances of free KIC (<6‐fold per ^1^H) are also observed in this NMR spectrum (see the Supporting Information).

**Figure 1:**
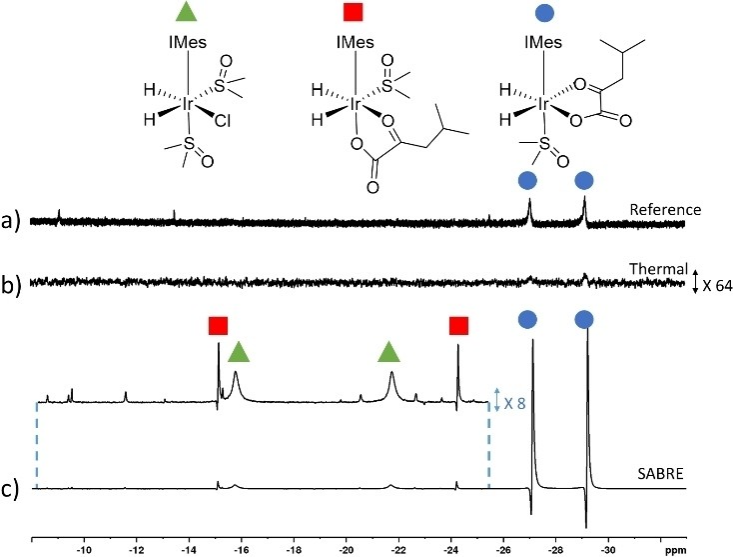
Partial a) 32 scan ^1^H NMR spectrum (not to scale) and b) corresponding single scan ^1^H NMR spectrum after a solution of [IrCl(COD)(IMes)] (5 mM), KIC (35 mM), dimethyl sulfoxide (22.5 mM) and 3 bar H_2_ are reacted in methanol‐*d*_4_ (0.6 mL) for *ca* 10 minutes. c) Corresponding SABRE hyperpolarized ^1^H NMR spectrum when this solution was shaken for 10 seconds with fresh *p*‐H_2_ at 6.5 mT. Note that b) has been vertically expanded relative to c) by a factor of 64, and the inset in c) has been expanded by a factor of 8.

These results suggest that a SABRE active polarization transfer catalyst has been created. This sample was then shaken at ∼ 1 μT in a mu metal shield for 10 seconds (SABRE‐SHEATH conditions).[^10^, ^17^] ^13^C NMR signals for free KIC could readily be discerned at *δ=*205.08 and 170.61 ppm which were enhanced by a factor of 160‐fold per ^13^C site (0.1 % ^13^C polarisation) when recorded at 9.4 T (Figure 2a). A hyperpolarized ^13^C signal, at *δ=*160.99 ppm, is also visible which is attributed to the carbene of the IMes ligand in [Ir(H)_2_(*κ* ^2^‐KIC)(NHC)(sulfoxide)].^[43]^ These results suggest that changing the pyruvate CH_3_ group to a CH_2_(CH_3_)_2_ group does not restrict the ability to form a SABRE active complex and therefore extension to a wider range of molecules with structural variety at this position could be possible.

**Figure 2:**
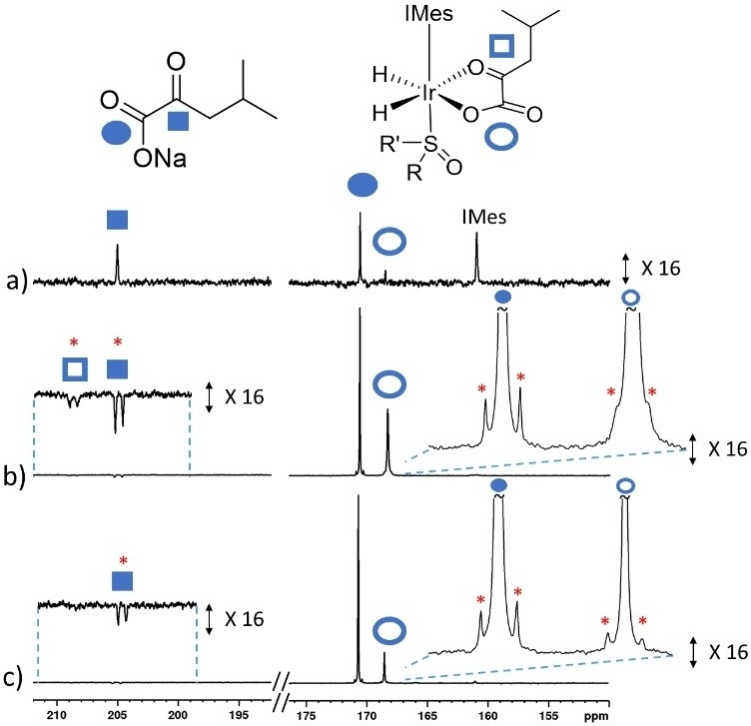
Partial hyperpolarized ^13^C NMR spectra when samples containing [IrCl(COD)(IMes)] (5 mM) and a) KIC and b)‐c) KIC‐1‐[^13^C] are shaken with a) dimethylsulfoxide (22.5 mM), b) dimethylsulfoxide (50 mM) and c) methylphenylsulfoxide (50 mM) and *p*‐H_2_ (3 bar) in methanol‐*d*_4_ (0.6 mL) for 10 seconds in a mu‐metal shield at *c.a*. 1 μT. Note the signals marked by a red asterisk denote singlet magnetization of naturally abundant KIC‐1,2‐[^13^C_2_].

We now extend these measurements to the hyperpolarization of ^13^C labelled material, KIC‐1‐[^13^C]. Two samples were prepared which contained [IrCl(COD)(IMes)] (5 mM), KIC‐1‐[^13^C] (35 mM) and either dimethylsulfoxide or methylphenylsulfoxide (50 mM) in methanol‐*d*_4_ (0.6 mL) as these were based on the conditions reported to give optimal ^13^C NMR signal enhancements for sodium pyruvate‐1‐2‐[^13^C_2_].^[16]^ ^1^H NMR measurements confirmed formation of the active polarization transfer catalyst, [Ir(H)_2_(*κ* ^2^‐KIC)(IMes)(sulfoxide)] in both cases. When these samples are shaken at ∼ 1 *μ*T in the mu metal shield ^13^C NMR signals are observed that arise from longitudinal single spin Zeeman magnetization of KIC‐1‐[^13^C] and two spin singlet order for the KIC‐1,2‐[^13^C_2_] isotopologue which is present at 1.1 % abundance (Figure 2b–c). The formation of singlet order within KIC‐1,2‐[^13^C_2_] as a consequence of SABRE is expected to be independent of polarization transfer field.[^43^, ^49^, ^50^] In contrast, the creation of single spin Zeeman magnetization is expected to be highly dependent on polarization transfer field.[^10^, ^13^, ^17^, ^44^] Therefore, the *p*‐H_2_ shaking process was increased to 20 seconds and repeated at various different magnetic fields (0.1 to 1.2 μT). These fields are achieved experimentally using a solenoid coil housed within a mu metal shield and has been described and used elsewhere.[^13^, ^43^] Higher SABRE performance is achieved when the direction of the field in the solenoid is aligned with that of the 9.4 T spectrometer. We note that similar field profiles, of lower magnitude, can be achieved when these fields align antiparallel to the 9.4 T field, which we ascribe to the sample passing through a zero‐field point during transfer between the shield and the spectrometer (see the Supporting Information).

The highest ^13^C NMR signal gains are achieved using polarization transfer fields of 0.7 μT and 0.5 μT when dimethylsulfoxide or methylphenylsulfoxide are used respectively (Figure 3). In each case, the SABRE ^13^C NMR signal gains are higher than those achieved by shaking at a ∼ 1 μT field for the same 20 second shaking time (955‐fold compared to 155‐fold and 985‐fold compared to 150‐fold when dimethylsulfoxide or methylphenylsulfoxide are used respectively). This is consistent with the need for a SABRE‐SHEATH matching condition to transfer polarization from *p*‐H_2_ derived hydride ligands directly to ligated ^13^C sites.[^10^, ^13^, ^17^, ^44^]

**Figure 3:**
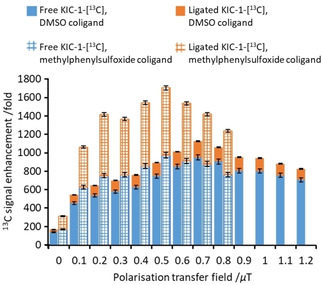
Delineated total ^13^C NMR signal enhancement for free (lower, blue) and bound (upper, orange) KIC‐1‐[^13^C] (35 mM) as a function of the polarization transfer field using precatalyst [IrCl(COD)(IMes)] (5 mM) and dimethylsulfoxide (solid bars) or methylphenylsulfoxide (grid bars) (50 mM) in methanol‐*d*_4_ (0.6 mL). Samples are shaken with *p*‐H_2_ for 20 seconds. These are single shot measurements with error bars calculated from typical values.^[16]^

While the maximum ^13^C NMR signal enhancement of free KIC‐1‐[^13^C] achieved using dimethylsulfoxide or methylphenylsulfoxide, at their optimal polarization transfer fields, are comparable (955‐fold compared to 985‐fold respectively), the ^13^C NMR signal enhancement for KIC‐1‐[^13^C] bound within the active polarization transfer catalyst is significantly higher when methylphenylsulfoxide is used (730‐fold compared to 175‐fold). This suggests that polarization transfer from *p*‐H_2_ into visible ^13^C magnetisation on ligated KIC‐1‐[^13^C] is more efficient within the active methylphenylsulfoxide‐containing catalyst compared to the dimethylsulfoxide analogue. However, greater polarisation losses during ligand dissociation from the methylphenylsulfoxide‐derived catalyst result in comparable ^13^C signal enhancements of KIC‐1‐[^13^C] free in solution for both catalysts. This difference is expected to be related to a range of factors including formation of a more efficient *J*‐coupled network,^[8]^ and faster relaxation within the active methylphenylsulfoxide‐derived catalyst, although ligand exchange and catalyst lifetime are also expected to play an important role.[^22^, ^36^]

These NMR samples were stable over the time‐period required to record the magnetic field profiles shown in Figure 3. However, when these methanol‐*d*_4_ solutions are left at room temperature for several hours, a significant drop in the resulting ^13^C NMR signal enhancements is observed upon performing SABRE with fresh *p*‐H_2_. Similar observations have been reported for pyruvate and related sulfoxide‐based catalysts and this has been attributed to catalyst deactivation.^[16]^

When the solution containing methylphenylsulfoxde is left for a period of several weeks at 278 K, growth of single crystals is observed. X‐ray diffraction of these reveals the presence of [Ir(*μ*‐H)(H)_2_(IMes)(SO(Ph)(Me)_2_)]_2_ (Figure 4a). Its structure is symmetric with both iridium centres exhibiting distorted octahedral geometries bridged by two hydride ligands. An iridium‐iridium bond length of 2.729 Å is consistent with reported values for iridium‐iridium dimers with metal‐metal single bonds (2.607 Å–2.826 Å).[^51^, ^52^, ^53^, ^54^] Shorter distances between iridium and the S sites of the sulfoxide (2.282 Å) support coordination through sulfur rather than oxygen (3.239 Å) and is consistent with related structures reported for similar systems.^[55]^ π stacking between the phenyl rings of the IMes ligand and the sulfoxide is expected to be an important interaction in this crystal (see the Supporting Information).

**Figure 4:**
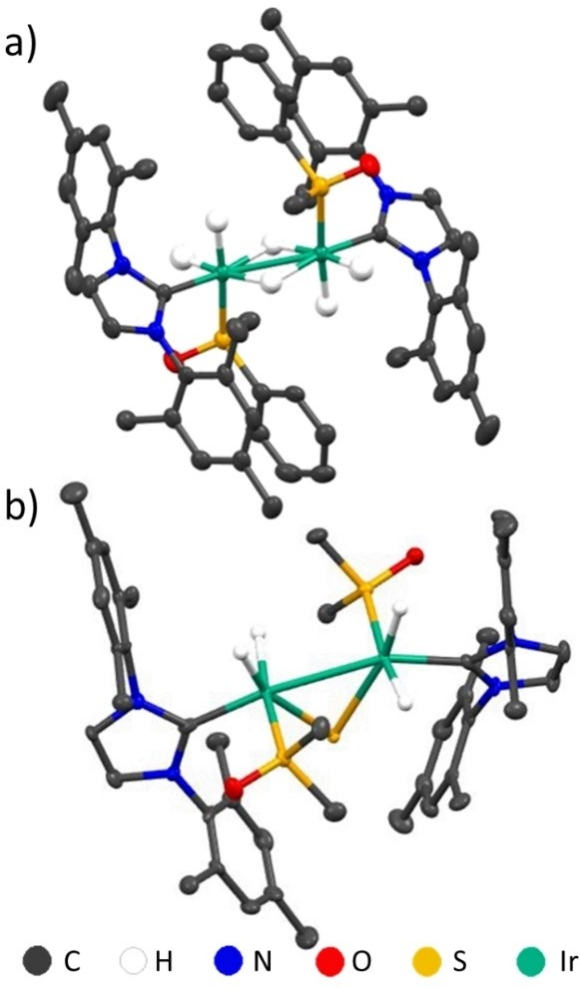
Single crystal X‐ray structures of a) [Ir(*μ*‐H)(H)_2_(IMes)(SO(Ph)(Me)_2_)]_2_ and b) [(Ir(H)_2_(IMes)(SO(Me)_2_))_2_(*μ*‐S)]. Solvents of crystallization and all non‐hydride hydrogen atoms have been omitted for clarity. Thermal ellipsoids are shown at 50 % probability. Refinement details are included in the Supporting Information.

The formation of single crystals is also observed when analogous solutions containing dimethylsulfoxde are left for a period of several weeks at 278 K. In this case, X‐ray diffraction confirms the presence of [(Ir(H)_2_(IMes)(SO(Me)_2_))_2_(*μ*‐S)] (Figure 4b). This structure also contains two distorted octahedral iridium centres, but they are now bridged by an S^2−^ ligand. The longer iridium‐iridium bond (2.908 Å) suggests a weaker metal‐metal interaction. Decomposition of phenylvinylsulfoxde and dibenzylsulfoxide to form related sulfur‐bridged iridium dimers has been observed in similar systems.^[55]^ We show here that this transformation can occur more generally and expect that this decomposition process will play a role in reduced SABRE efficiency of such catalytic systems at long reaction times. Formation of oligomeric products from Ir‐based catalysts, and their effect on reducing SABRE efficiency have been reported previously.[^32^, ^56^]

In conclusion, we demonstrate that the scope of sulfoxide‐containing SABRE polarization transfer catalysts can be extended to hyperpolarize molecules with similar α‐keto acid motifs to pyruvate. This could lead to a larger range of potential applications of SABRE in monitoring chemical transformations. For example, KIC is a metabolic precursor to amino acids such as leucine and has been used as a hyperpolarized reporter to study this transformation *in vivo*.[^46^, ^47^, ^48^]

The largest ^13^C NMR signal gains achieved for KIC‐1‐[^13^C] in this work are 985‐fold (0.8 % polarization) which is lower than those achieved using alternative hyperpolarization techniques such as dissolution dynamic nuclear polarization (d‐DNP) (15–32 % polarization).[^46^, ^47^, ^48^] In contrast, SABRE uses a simple, refreshable set‐up and achieves these enhanced signals in a 20 second polarization step which is significantly shorter than the 1–1.5 hours required using d‐DNP.^[46]^ It is expected that these SABRE signal gains can be increased by further optimization of factors including temperature, sulfoxide, carbene ligand, *p*‐H_2_ pressure, temperature, and many others.[^10^, ^12^, ^16^, ^17^, ^18^, ^19^, ^20^, ^21^, ^22^] We expect the types of molecules whose MR signals these catalysts can sensitize to increase in the future.

## Conflict of interest

The authors declare no conflict of interest.

## Supporting information

As a service to our authors and readers, this journal provides supporting information supplied by the authors. Such materials are peer reviewed and may be re‐organized for online delivery, but are not copy‐edited or typeset. Technical support issues arising from supporting information (other than missing files) should be addressed to the authors.

Supplementary
