# Supplementary material for: Reversible Hyperpolarization of Ketoisocaproate Using Sulfoxide‐containing Polarization Transfer Catalysts
Source: Chemphyschem. 2020 Nov 26;22(1):13–7. doi: 10.1002/cphc.202000825 (PMC7839500; doi:10.1002/cphc.202000825)
Supplement: Supplementary file 1 — Supplementary [file CPHC-22-13-s001.pdf]

ChemPhysChem

Supporting Information

Reversible Hyperpolarization of Ketoisocaproate Using Sulfoxide-containing Polarization Transfer Catalysts

Ben. J. Tickner, Fadi Ahwal, Adrian C. Whitwood, and Simon B. Duckett*

Supporting Information

Table of Contents

S1: General remarks
S2: Hyperpolarized NMR spectra
S3: X-ray crystallography
S4: References

S1: General remarks

All NMR measurements were carried out on a 400 MHz Bruker Avance III spectrometer at room temperature (298 K) unless otherwise stated. *Para*-hydrogen (p-H_2) was produced by passing hydrogen gas over a spin-exchange catalyst (Fe_2O_3) at 28 K and used for all the hyperpolarization experiments. This method produces *p*-H_2 with ca. 98% purity. ^{1}H (400 MHz) and ^{13}C (100.6 MHz) NMR spectra were recorded with an internal deuterium lock. Chemical shifts are quoted as parts per million and referenced to residual methanol-d_3 as the solvent. ^{13}C NMR spectra were recorded without broadband proton decoupling. Coupling constants (*J*) are quoted in Hertz.

The shake & drop method was employed for recording hyperpolarized NMR spectra. Samples were prepared containing [IrCl(COD)(IMes)] (where IMes = 1,3-bis(2,4,6-trimethylphenyl)imidazole-2-ylidene and COD = *cis*,*cis*-1,5-cyclooctadiene) (5 mM) and the indicated sulfoxide with sodium ketoisocaproate (35 mM, 7 eq.) or sodium ketoisocaproate-1-[^{13}C] (35 mM, 7 eq.) in 0.6 mL of methanol-d_4 in a 5 mm NMR tube that was fitted with a J. Young's tap. The iridium catalyst was synthesized according to a literature procedure.[1] All the commercially available compounds used were purchased from Sigma-Aldrich, Fluorochem or Alfa-Aesar and used as supplied. The solutions were subsequently degassed by two freeze-pump-thaw cycles before filling the tube with *p*-H_2 at 3 bar pressure. Once filled with *p*-H_2, the tubes were shaken vigorously for the indicated time (10 or 20 seconds) in either, a) the 65 Gauss fringe field of a 9.4 T Bruker spectrometer for polarization transfer to ^{1}H nuclei, or b) mu metal shield (~1 µT) or c) a home-built mu metal shielded solenoid (fields between 0.1 µT and 1.2 µT) for transfer to ^{13}C nuclei (SABRE-SHEATH conditions). Immediately after shaking, the NMR tubes were placed rapidly inside the spectrometer for NMR detection.

NMR signal enhancements were calculated by dividing the hyperpolarized signal intensity by the corresponding signal intensity in a thermal spectrum. The thermal and hyperpolarized spectra are recorded using the same acquisition and spectral parameters and the thermal sample was left inside the magnet for sufficient time to allow equilibration with the spectrometer magnetic field. In the case of ^{13}C signal enhancements for unlabelled ketoisocaproate, these were estimated with reference to the thermally polarized methanol solvent signal using an adapted previously described equation[2] as thermal signals for ketoisocaproate could not be discerned in a single scan ^{13}C NMR measurement.

S2: Hyperpolarized NMR spectra

Ketoisocaproate (KIC) (35 mM, 7 eq.) was activated with [IrCl(COD)(IMes)] (5 mM), dimethyl sulfoxide (22.5 mM, 4.5 eq.) and 3 bar H_2 in methanol-d_4 (0.6 mL). Upon shaking this solution for 10 seconds with *p*-H_2 (3 bar) at 6.5 mT weakly hyperpolarized signals for the ^{1}H resonances of KIC (< 6-fold per ^{1}H) are observed, as shown in Figure S1.

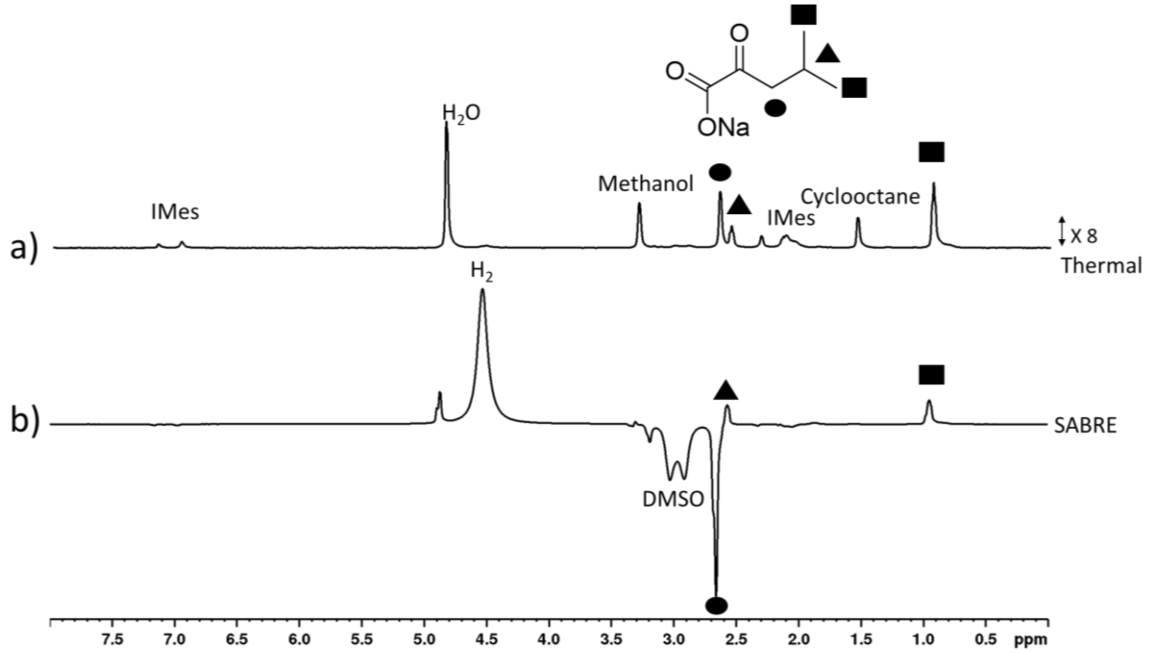

Figure S1. Partial a) single scan ^{1}H NMR spectrum and b) corresponding SABRE hyperpolarized ^{1}H NMR spectrum after a solution of [IrCl(COD)(IMes)] (5 mM), KIC (35 mM, 7 eq.), dimethyl sulfoxide (22.5 mM, 4.5 eq.) and 3 bar *p*-H_2 in methanol-d_4 (0.6 mL) is shaken for 10 seconds at 6.5 mT. Note that spectrum a) has been vertically expanded relative to spectrum b) by a factor of 8.

Hyperpolarized hydride signals were compared in systems containing ^{13}C labelled material, KIC-1-[^{13}C]. Two samples were prepared containing [IrCl(COD)(IMes)] (5 mM), KIC-1-[^{13}C] (35 mM, 7 eq.) and either dimethylsulfoxide or methylphenylsulfoxide (50 mM, 10 eq.) in methanol-d_4 (0.6 mL). They were compared to a sample containing unlabelled ketoisocaproate (KIC) (35 mM, 7 eq.), [IrCl(COD)(IMes)] (5 mM), dimethyl sulfoxide (22.5 mM, 4.5 eq.) and 3 bar H_2 in methanol-d_4 (0.6 mL). Each solution was shaken for 10 seconds with p-H_2 (3 bar) at 6.5 mT and the spectra are shown in Figure S2.

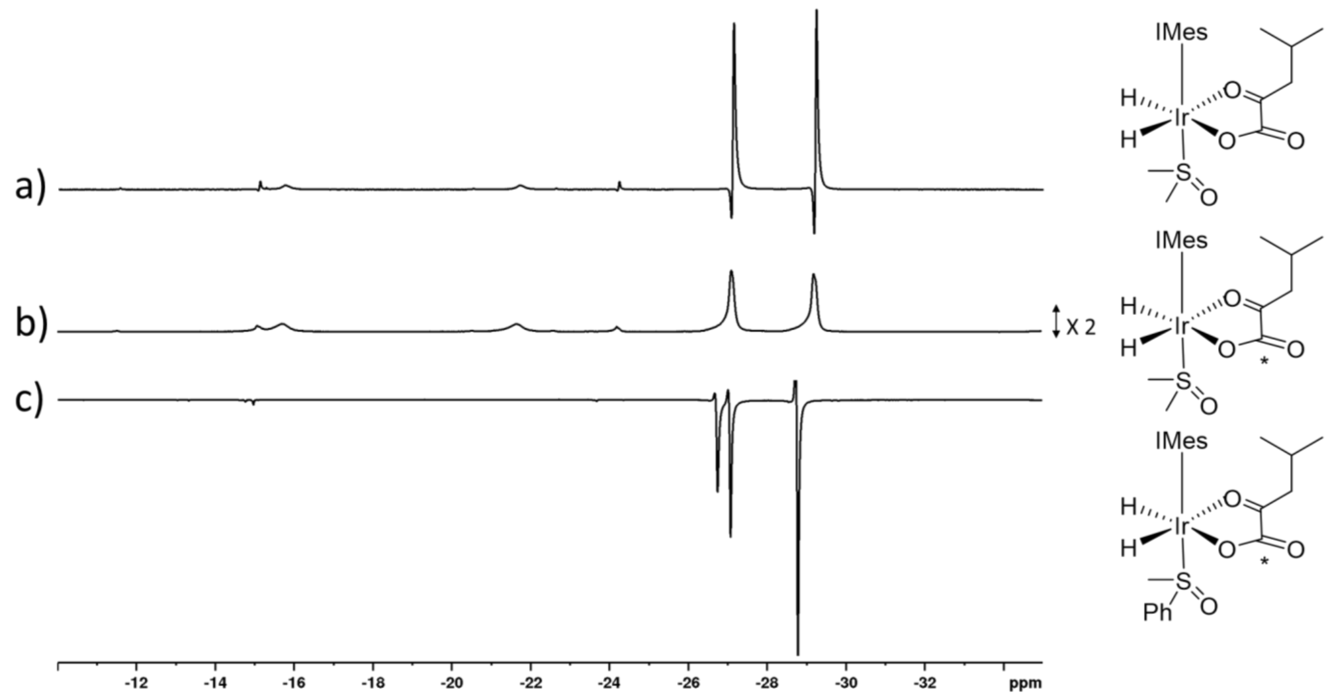

***Figure S2**.* Partial hyperpolarized ^{1}H NMR spectra when samples containing [IrCl(COD)(IMes)] (5 mM) and a) KIC and b)-c) KIC-1-[^{13}C] (35 mM, 7 eq.) are shaken with a) dimethylsulfoxide (22.5 mM, 4.5 eq.), b) dimethylsulfoxide (50 mM, 10 eq.) and c) methylphenylsulfoxide (50 mM, 10 eq.) and p-H_2 (3 bar) in methanol-d_4 (0.6 mL) for 10 seconds in a mu-metal shield at *c.a*. 1 µT.

The effect of magnetic field on ^{13}C NMR signal enhancements for the samples containing KIC-1-[^{13}C] was investigated. These fields are achieved experimentally using a mu metal shield housed within a solenoid coil. Lower SABRE performance is observed when the direction of the field in the solenoid is aligned against the 9.4 T spectrometer field which is linked to passing through a zero field point upon transfer between shield and spectrometer. The ^{13}C NMR signal enhancement field dependancy when the shield field was alligned against the spectrometer is shown in Figure S3. (For the plot presented in the manuscript the two fields are alligned parallel).

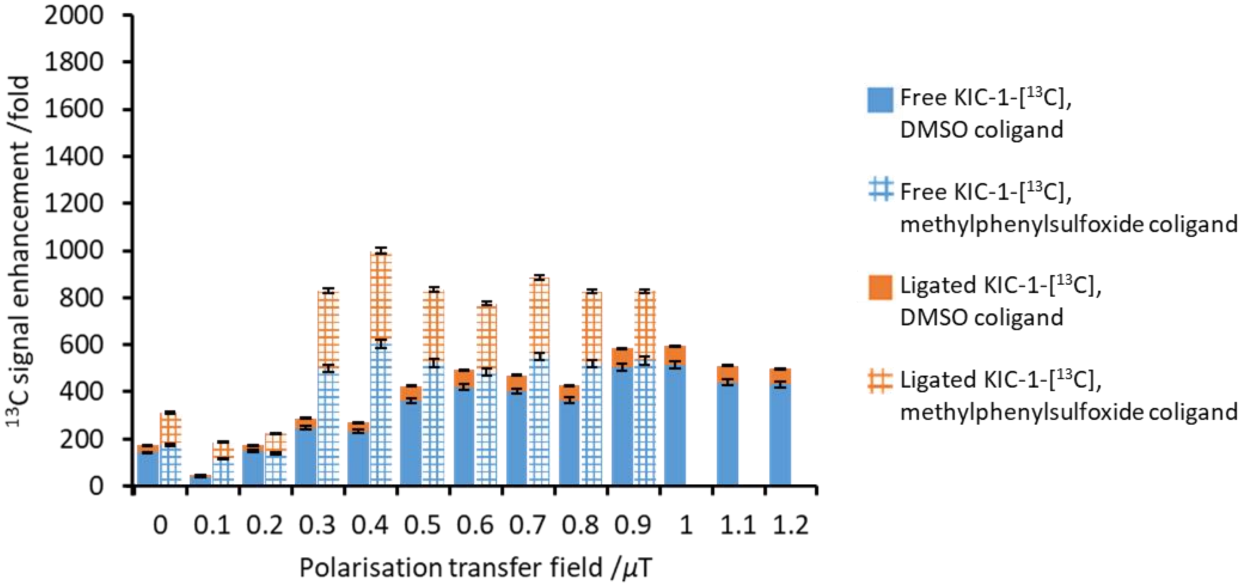

Figure S3. Dependence of ^{13}C NMR signal enhancement of free (lower, blue) and bound (upper, orange) KIC-1-[^{13}C] (35 mM, 7 eq.) on the polarization transfer field for samples containing [IrCl(COD)(IMes)] (5 mM) and either dimethylsulfoxide (solid bars) or methylphenylsulfoxide (grid bars) (50 mM, 10 eq.) in methanol-d_4 (0.6 mL) shaken with *p*-H_2 for 20 seconds in a mu-metal shielded solenoid. The data are single shot measurements with error bars set to those of a typical sample.[3]

S3: X-ray crystallography

[Ir(μ-H)(H)$_2$(IMes)(SO(Ph)(Me)$_2$)]$_2$ and [(Ir(H)$_2$(IMes)(SO(Me)$_2$))$_2$(μ-S)] were prepared by leaving a solution of [IrCl(COD)(IMes)] (5 mM), sodium ketoisocaproate-1-[^{13}C] (35 mM, 7 eq.) and methylphenylsulofxide or dimethylsulfoixde (50 mM, 10 eq.) respectively with 3-bar H_2 in methanol-d_4 at 278 K for a period of several weeks.

For X-ray diffraction studies, a suitable crystal was selected and mounted on an Oxford Diffraction SuperNova- X-ray diffractometer. The crystal was kept at 110 K during data collection. Diffractometer control, data collection, initial unit cell determination, frame integration and unit-cell refinement was carried out with "CrysAlisPro".[4] Face-indexed absorption corrections were applied using spherical harmonics, implemented in SCALE3 ABSPACK scaling algorithm. Using Olex2,[5] the structure was solved with the ShelXT[6] structure solution program using Intrinsic Phasing and refined with the ShelXL[7] refinement package using Least Squares minimization.

The crystals of [Ir(μ-H)(H)$_2$(IMes)(SO(Ph)(Me)$_2$)]$_2$ contained highly disordered methanol in channels running parallel to the C-axis. A discrete atom model could not be obtained so these were modelled using a solvent mask. This calculated that there was a single void per unit-cell with a volume of 237 Å^3 containing 45 electrons which corresponds to 2.5 molecules of methanol. The structure was deposited on the CCDC (deposition number 2023460).

The hydrides of [(Ir(H)$_2$(IMes)(SO(Me)$_2$))$_2$(μ-S)] were initially located by an electron density difference map and subsequently the Ir-H bond lengths were fixed at 1.65 Å. The structure was deposited on the CCDC (deposition number 2023461).

An alternative orientation of [Ir(μ-H)(H)$_2$(IMes)(SO(Ph)(Me)$_2$)]$_2$ to that presented in the main manuscript is shown in Figure S4 to highlight π stacking interactions. Key X-ray crystallography details are presented in Table S1.

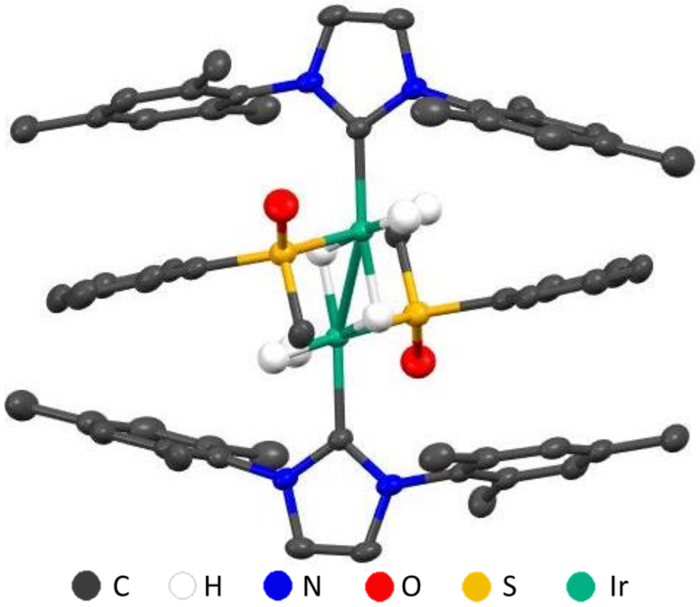

Figure S4. Structure of [Ir(μ-H)(H)$_2$(IMes)(SO(Ph)(Me)$_2$)]$_2$ determined from single crystal X ray crystallography. Solvents of crystallization and all non-hydride hydrogen atoms have been omitted for clarity. Thermal ellipsoids are shown at 50% probability.

Table S1. Crystal data and structure refinement for $[Ir(\mu\text{-}H)(H)_2(IMes)(SO(Ph)(Me)_2)]_2$ and $[(Ir(H)_2(IMes)(SO(Me)_2))_2(\mu\text{-}S)]$

Compound	$[Ir(\mu\text{-}H)(H)_2(IMes)(SO(Ph)(Me)_2)]_2$	$[(Ir(H)_2(IMes)(SO(Me)_2))_2(\mu\text{-}S)]$
Empirical formula	$C_{58}H_{78}Ir_2N_4O_4S_2$	$C_{46}H_{64}Ir_2N_4O_2S_3$
Formula weight	1343.76	1185.59
Temperature/K	110.00(10)	110.00(10)
Crystal system	triclinic	orthorhombic
Space group	P-1	$Pna2_1$
a/Å	11.0591(6)	16.5217(3)
b/Å	12.0198(7)	11.6762(2)
c/Å	13.1523(7)	24.9575(5)
α/°	98.154(5)	90
β/°	107.277(5)	90
γ/°	106.939(5)	90
Volume/$Å^3$	1546.29(16)	4814.60(15)
Z	1	4
$\rho_{calc}g/cm^3$	1.443	1.636
μ/mm^{-1}	9.178	12.062
F(000)	672.0	2344.0
Crystal size/mm^3	0.169 × 0.112 × 0.095	0.142 × 0.098 × 0.077
Radiation	CuKα (λ = 1.54184)	CuKα (λ = 1.54184)
2Θ range for data collection/°	7.27 to 134.112	7.084 to 134.15
Index ranges	-13 ≤ h ≤ 13, -14 ≤ k ≤ 12, -15 ≤ l ≤ 15	-14 ≤ h ≤ 19, -10 ≤ k ≤ 13, -29 ≤ l ≤ 28
Reflections collected	10218	10987
Independent reflections	5537 [R_{int} = 0.0362, R_{sigma} = 0.0527]	6963 [R_{int} = 0.0204, R_{sigma} = 0.0318]
Data/restraints/parameters	5537/1/336	6963/1/531
Goodness-of-fit on F^2	1.004	1.044
Final R indexes [I>=2σ (I)]	R_1 = 0.0328, wR_2 = 0.0769	R_1 = 0.0247, wR_2 = 0.0556
Final R indexes [all data]	R_1 = 0.0387, wR_2 = 0.0796	R_1 = 0.0309, wR_2 = 0.0594
Largest diff. peak/hole / e $Å^{-3}$	1.86/-0.90	0.94/-0.67

S4: References

[1] L. D. Vazquez-Serrano, B. T. Owens, J. M. Buriak, *Inorg. Chim. Acta* **2006**, *359*, 2786-2797.
[2] B. J. Tickner, W. Iali, S. S. Roy, A. C. Whitwood, S. B. Duckett, *ChemPhysChem* **2019**, *20*, 241-245.
[3] B. J. Tickner, O. Semenova, W. Iali, P. J. Rayner, A. C. Whitwood, S. B. Duckett, *Cat. Sci. Technol.* **2020**, *10*, 1343-1355.
[4] Oxford. Diffraction. Ltd., **Version 1.171.34.41**.
[5] O. V. Dolomanov, L. J. Bourhis, R. J. Gildea, J. A. Howard, H. Puschmann, *J. Appl. Cryst.* **2009**, *42*, 339-341.
[6] G. Sheldrick, *Acta Crys. Sec. A.* **2015**, *71*, 3-8.
[7] G. Sheldrick, *Acta Crys. Sec. C.* **2015**, *71*, 3-8.
